# Plasma Levels of Pentosidine, Carboxymethyl-Lysine, Soluble Receptor for Advanced Glycation End Products, and Metabolic Syndrome: The Metformin Effect

**DOI:** 10.1155/2016/6248264

**Published:** 2016-10-18

**Authors:** Mohamed Haddad, Ines Knani, Hsan Bouzidi, Olfa Berriche, Mohamed Hammami, Mohsen Kerkeni

**Affiliations:** ^1^Laboratory of Biochemistry (LR12ES05) “Nutrition-Aliment Fonctionnel et Santé vasculaire”, Faculty of Medicine, University of Monastir, Monastir, Tunisia; ^2^Laboratory of Biochemistry, CHU Thar Sfar, Mahdia, Tunisia; ^3^Laboratory of Endocrinology, CHU Thar Sfar, Mahdia, Tunisia

## Abstract

Metabolic syndrome (MetS) is considered one of the most important public health problems. Several and controversial studies showed that the role of advanced glycation end products (AGEs) and their receptor in the development of metabolic syndrome and therapeutic pathways is still unsolved. We have investigated whether plasma pentosidine, carboxymethyl-lysine (CML), and soluble receptor for advanced glycation end products (sRAGE) levels were increased in patients with MetS and the effect of metformin in plasma levels of pentosidine, CML, and sRAGE. 80 control subjects and 86 patients were included in this study. Pentosidine, CML, and sRAGE were measured in plasma by enzyme-linked immunosorbent assay (ELISA). Plasma pentosidine, CML, and sRAGE levels were significantly increased in patients compared to control subjects (*P* < 0.001, *P* < 0.001, and *P* = 0.014, resp.). Plasma levels of pentosidine were significantly decreased in patients who received metformin compared to untreated patients (*P* = 0.01). However, there was no significant difference between patients treated with metformin and untreated patients in plasma CML levels. Plasma levels of sRAGE were significantly increased in patients who received metformin and ACE inhibitors (*P* < 0.001 and *P* = 0.002, resp.). However, in a multiple stepwise regression analysis, pentosidine, sRAGE, and drugs treatments were not independently associated. Patients with metabolic syndrome showed increased levels of AGEs such as pentosidine and CML. Metformin treatment showed a decreased level of pentosidine but not of CML. Therapeutic pathways of AGEs development should be taken into account and further experimental and* in vitro* studies merit for advanced research.

## 1. Introduction

Cardiovascular diseases are one of the main causes of mortality in industrialized countries, due to an increase in prevalence of different cardiovascular risk such as abdominal obesity, insulin resistance, dyslipidemia, hypertension, and hyperglycemia. These metabolic disorders presented within the same individual give rise to metabolic syndrome. Recent estimates are that between 20 and 30% of the adult population worldwide have a metabolic syndrome [1]. The causes of this disease are multifactorial, stemming from complex genetic and environmental influences such as physical inactivity, smoking, and a diet rich in sugars and saturated fatty acids.

Due to complex metabolic disorder in metabolic syndrome, reactive derivatives are formed via nonenzymatic reaction named Maillard reaction between the free amino groups in lysine and arginine residues in proteins and carbohydrates that undergo a series of complex reactions to an irreversible complex group of compound termed advanced glycation end products (AGEs) [2, 3]. These products can be formed by covalent binding of methylglyoxal (MG) and glyoxal to the free amino and thiol groups of proteins [4]. In addition to their endogenous formation, AGEs exist in high amounts in cooked fast-food diet and known by brown coloration, but only a small quantity of these compounds present in food are absorbed and the other part is secreted by the kidneys in urine [5]. However, pentosidine and *ε*-N-carboxymethyl-l-lysine (CML) have been well characterized as biomarkers for the formation and accumulation of AGEs [6] and are known to play an important role in diabetes and vascular complications [7–9]. AGEs cause damage by affecting protein structure, by formation of cross-links between molecules, or by binding the receptor for AGEs (RAGE) [10–12]. RAGE is a member of the immunoglobulin superfamily of cell surface proteins that binds AGEs and other molecules.

Previous studies have confirmed that interaction between AGEs and RAGE activates the generation of oxidative stress and production of proinflammatory cytokines in various types of cells that have been implicated in metabolic and many other disease statuses [13–16]. Furthermore, Monden et al. have shown that RAGE affects adipocyte hypertrophy and insulin sensitivity in mice [17]. In addition to the membrane form, RAGE is found in the soluble form (sRAGE) and two different types are identified. sRAGE formed by the cleavage of RAGE by metalloproteinases ADAM10 and MMP9 [18]. esRAGE (endogenous secretory RAGE) formed by the alternative splicing of RAGE mRNA. Because of its ability to bind with the same ligands, sRAGE acts as a competitive inhibitor of RAGE and precludes the cell-bound RAGE signaling [19]. For this reason, many clinical studies have concluded the possibility of using sRAGE as a disease marker in such disorders [20, 21]. Several and controversial studies showed that the roles of AGEs and their receptor in the development of metabolic syndrome and therapeutic pathways to decrease the level of these biomarkers are still unsolved.

The present study was designed to elucidate, in the first part, the level of serum pentosidine, CML, and soluble form of RAGE (sRAGE) in patients with metabolic syndrome and, in the second part, to investigate the effect of several drugs, in particular, metformin which is known by its antiglycation effect, on pentosidine, CML, and sRAGE levels in these patients.

## 2. Subjects and Methods

### 2.1. Subjects

This study included 80 control subjects and 86 patients who developed a metabolic syndrome recruited from Department of Endocrinology of Taher Sfar Hospital in Mahdia (Tunisia). The criteria of the American Heart Association Scientific Statements of 2009 were used to define metabolic syndrome [22]. Patients were considered to have metabolic syndrome if they presented 3 or more of the following risk factors: (1) elevated waist circumference (≥90 cm for men and ≥80 cm for women); (2) elevated TG (≥1.7 mM) (drug treatment for elevated triglycerides is an alternate indicator); (3) reduced HDL-C (<1 mM for men and <1.3 mM for women) (drug treatment for reduced HDL-C is an alternate indicator); (4) elevated blood pressure (systolic BP (SBP) ≥130 mmHg or diastolic BP (DBP) ≥85 mmHg) (antihypertensive drug treatment in a patient with a history of hypertension is an alternate indicator); and (5) elevated fasting glucose (≥5.5 mM) (drug treatment of elevated glucose is an alternate indicator). Data collection was performed using an information sheet, specifically designed and containing all the personal information as well as the various elements of metabolic syndrome. For this, a clinical examination was carried out to determine the weight, height, waist circumference, blood pressure, and the drug treatments from each patient. In fact, patients were divided into patients who are treated with metformin (Glucophage 850 mg; 3 times/day) (*n* = 55) and patients who are untreated with this drug (*n* = 31).

### 2.2. Methods

In all subjects, venous blood was collected in the morning after an overnight fast. After centrifuging the samples at 3000 rpm for 10 min at 4°C, plasma and serum were collected and stored at −80°C until analysis. Fasting plasma glucose, triglyceride, total cholesterol, HDL cholesterol, and LDL cholesterol were measured using enzymatic methods by CX9 autochemical analysis instrument (Bechman CX9, USA). Hemoglobin A1C (HbA1c) was measured using G7 HPLC Analyser (Tosoh Europe NV). Plasma levels of pentosidine, CML, and sRAGE were determined by enzyme-linked immunosorbent assay (ELISA) provided by Cusabio Biotech Co., Ltd., according to the manufacturer's instructions. Detection range of pentosidine CML and sRAGE kit was, respectively, 31–2000 pmol/mL, 62–4000 pg/mL, and 78–5000 pg/mL. The intra-assay and interassay coefficients of variation were <8% and <10%, respectively.

### 2.3. Statistical Analysis

The study data was expressed as mean ± SD or median and interquartile ranges (IQR) using the SPSS program (version 18). Differences between groups were analyzed by the independent sample Student's* t*-test or Mann–Whitney *U* test. A *P* value less than 0.05 was considered statistically significant. Correlation was determined by a linear regression analysis and a multiple regression analysis was used to further explore the linear relationships between the variables. Regression variables were estimated as well as the correlation coefficient *r*. ANOVA was used to assess the significance of the regression with significance accepted at *P* < 0.05.

## 3. Result

### 3.1. Clinical and Anthropometric Characteristics

The clinical, anthropometric characteristics and treatment drugs of controls and patients with MetS are shown in Table 1. No difference between the mean age of control subjects and patients was found. In total, 86% of patients were diabetic, 69.8% had hypertension, and 33.7% had hyperlipidaemia. Significant differences were seen between the groups in waist circumference, body mass index, serum glucose, HbA1c, triglyceride (*P* < 0.001), and total and HDL cholesterol (*P* < 0.01).

### 3.2. Pentosidine, CML, and sRAGE Levels in Control Subjects and Patients with MetS

Table 2 shows results of plasma pentosidine, CML, and sRAGE levels in patients and control subjects. Patients showed an increased level of pentosidine, CML, and sRAGE compared to control subjects (211.21 (69.94–379.89) versus 53.15 (36.61–60.34) pmol/mL; 440.38 (383.98–601.36) versus 171.75 (81.03–298.06) pg/mL; 155 (126.34–240.4) versus 117.33 (104.38–158.04) pg/mL; *P* < 0.001, *P* < 0.001, and *P* = 0.014, resp.).

### 3.3. Treatment Effect of Metformin on Pentosidine, CML, and sRAGE Levels in Patients with MetS

**Figure 1:**
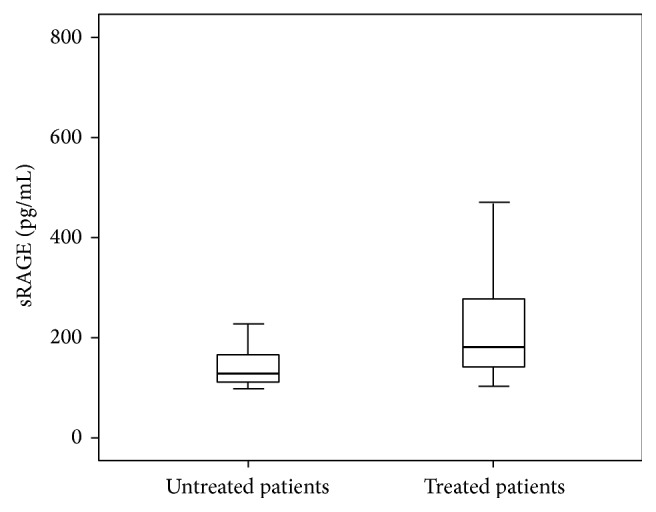
Box plots of plasma sRAGE levels in treated (*n* = 55) and untreated (*n* = 31) patients with metformin. The horizontal lines in each box represent (bottom to top) the 10th, 25th, 50th (median), 75th, and 90th percentiles. sRAGE: soluble receptor for AGE (*P* < 0.001).

**Figure 2:**
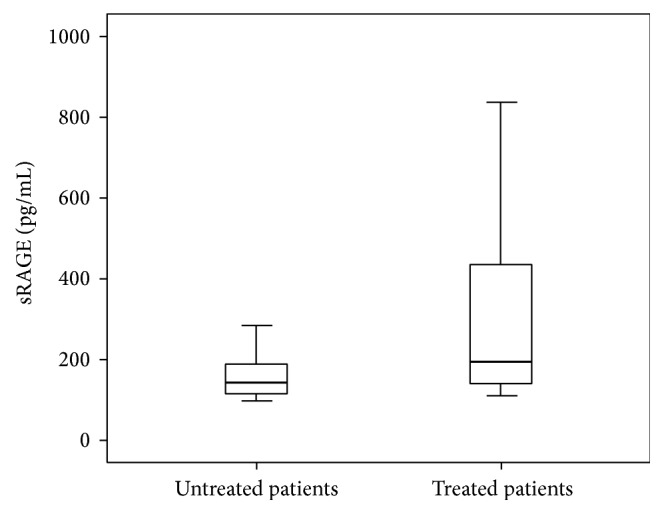
Box plots of plasma sRAGE levels in treated (*n* = 33) and untreated (*n* = 53) patients with ACE inhibitors (*P* = 0.002).

As shown in Table 3, the plasma levels of pentosidine were significantly decreased between patients who received metformin and patients without this treatment: 140.72 (62.07–249.51) versus 372.45 (132.55–556.68) pmol/mL (*P* = 0.01), respectively. However, no significant difference between metformin effects on plasma CML levels had been shown. Plasma levels of sRAGE were significantly increased between patients who received metformin and patients without this treatment: 180.9 (142.33–273.85) versus 131.84 (111.75–226.98) pg/mL (*P* < 0.001), respectively. We cannot conclude that this increase is due to the effect of metformin because there is an increase in sRAGE plasma levels in patients who are treated with ACE inhibitors: 194.9 (138.59–468.59) versus 142.33 (116.69–194.32) pg/mL in untreated patients (*P* = 0.002). This finding was illustrated in Figures 1 and 2. However, no significant difference between treated patients and untreated patients with other drugs on plasma pentosidine and CML levels had been shown.

### 3.4. Linear Regression and Multivariate Analyses

A linear regression analysis in patients with MetS showed negative and significant correlation between pentosidine levels and metformin treatment. sRAGE levels correlated positively with metformin treatment and/or ACE inhibitors treatment. However, no significant correlation between drugs treatments and CML levels had been shown (Table 4). To explain the levels variance of pentosidine and sRAGE in patients who received metformin, multiple linear regression model was formed. This model, which was applied to different drugs treatment (considered as independent variables) as well as pentosidine and sRAGE as the dependent variable, showed that these two biomarkers are not correlated positively to the treatment (Table 5).

## 4. Discussion

Predictors and identification of factors involved in the modulation of metabolic disorders of metabolic syndrome may be important in the prevention and the management of their complications. For this context, in the first part, the present study demonstrated new biomarkers such as plasma pentosidine, CML, and sRAGE levels, which are increased in patients with MetS. In the second part, our study evaluated the levels of these biomarkers in patients treated with drugs recognized by their antiglycation effect as metformin. Our results showed decreased pentosidine and increased sRAGE plasma levels in treated patients compared with untreated patients.

A number of clinical studies have reported that AGEs accumulate at a much higher rate in diabetic than in normal population [23–25]. Other studies have shown that circulating levels of AGEs are positively associated with inflammatory markers, endothelial dysfunction, insulin resistance, and vascular complications in diabetes [26–29]. Serum levels of pentosidine were found to be significantly higher in patients with diabetes than those without diabetes. Moreover, pentosidine levels were significantly higher in diabetic patients with cardiovascular disease than in those without cardiovascular disease [30, 31]. Kerkeni et al. found that pentosidine is a biomarker for microvascular complications in type 2 diabetic patients and an independent determinant of the presence of hypertension and hyperlipidaemia [8]. Indicators of the degree of vascular damage, on cardiovascular disease, are dramatically amplified when diabetes is added to dyslipidemia, hypertension, and obesity. Our study showed that pentosidine levels were markedly increased in patients with MetS compared with our previous studies. However, no specific biomarkers have been studied to resolve the tsunami effect of metabolic syndrome, as a multifactorial disease. In addition to the interest effects of pentosidine, many studies have reported that CML serum levels are higher in diabetic patients compared to nondiabetic patients [32–34]. Other results have showed a positive association between serum CML and vascular complication in diabetes [35–37]. According to these results, our study showed increased plasma levels of CML in patients with MetS compared to control subjects.

The reason why AGEs are involved in the genesis of metabolic syndrome needs to be explained. At the molecular levels, AGEs including pentosidine and CML cause damage by two principal mechanisms. First, structural modification of proteins perturbs their normal function. Second, AGEs are ligands for the receptor for AGEs (RAGE) causes intracellular oxidative stress and activation of nuclear factor NF-K*β* who modulates genes transcription for various factors: endothelin-1, vascular endothelial growth factor (VEGF), transforming growth factor-*β* (TGF-*β*), proinflammatory cytokines such as interleukins 1 and 6, and tumor necrosis factor-*α* (TNF-*α*) [38, 39]. As a consequence, AGEs are related to wide variety of systemic pathologic conditions including ageing, hypertension, renal failure, diabetes, or cardiovascular complications [40].

A several metabolic components well established as risk factors for cardiovascular disease have also been shown to be associated with altered plasma sRAGE or esRAGE. Plasma esRAGE levels are decreased in patients with MetS and are inversely correlated with several components of MetS including body mass index, blood pressures, insulin resistance index, fasting plasma glucose, serum triglyceride, and lower HDL cholesterol levels [41]. Hudson et al. in the Northern Manhattan study demonstrated that lower sRAGE levels are not only associated with MetS but also lower in proportion to the number of metabolic components [42]. The findings regarding the soluble form of RAGE in diabetes are quite confusing. The same groups have found that plasma esRAGE and sRAGE levels are significantly lower in type 1 and type 2 diabetic patients than in nondiabetic controls [43, 44]. Other groups found that plasma sRAGE levels increased in type 1 and type 2 diabetic patients [45–47]. This confusion in the plasma sRAGE levels may be due to the treatment effect. sRAGE levels are affected by drugs such as ACE inhibitors, and patients treated with this drugs have significantly higher levels of circulating sRAGE [48]. Our study demonstrated that plasma levels of sRAGE were significantly increased in treated patients with metformin compared to untreated patients but we cannot conclude that this increase is due to the effect of metformin because there is an increase in sRAGE plasma levels in patients treated with ACE inhibitors.

In addition to its action on glycemic control through the decrease in hepatic glucose production and increasing the sensitivity of peripheral cells to insulin, a number of studies have shown that metformin is beneficial in reducing diabetes associated vascular risk by inhibition of glycation process and the intermediate of AGEs such as MG [49, 50]. The chemical structure of this drug is related to that of aminoguanidine which could be the highlight for inhibiting the formation of AGEs [51]. Diamanti-Kandarakis et al. have shown that plasma AGEs levels were reduced after 6 months of metformin administration in women with polycystic ovary syndrome [52]. Other studies have confirmed that metformin can reduce AGEs levels in lenses, kidneys, and nerves in diabetic animals [53]. According to these results, our study showed a decreased levels of pentosidine but not CML in patients who are treated with metformin. The mechanism by which metformin lowers plasma levels of pentosidine and not those of CML in the studied group of MetS is not clear; however, there is evidence that its effect could interfere with formation pathway. Pentosidine could be formed by the reaction of ribose, glucose, fructose, or ascorbate with lysine and arginine [54]. Particularly, CML could be formed by the reaction of glucose and other sugars and glyoxal with amino groups of arginine and lysine or by lipids peroxidation [55]. So, the rate of CML formation in patients can be dominated by the inhibition capacity of metformin.

## 5. Conclusion

Patients with metabolic syndrome showed increased levels of AGEs products such as pentosidine and CML. Metformin treatment effect showed a decreased level of pentosidine but not of CML. Therapeutic ways of AGEs development should be taken into account and further experimental and* in vitro* studies merit for advanced research.

## Figures and Tables

**Table 1:** Clinical and anthropometric parameters of control subjects and patients with MetS.

Characteristic	Control subjects *n* = 80	Patients *n* = 86	*P*
Sex (M/F)	(41/39)	(40/46)	NS
Age (years)	57 ± 11	59 ± 12	NS
Waist circumference (cm)	84.5 ± 5.4	109.8 ± 13.4	<0.001
BMI (kg/m^2^)	20.8 ± 1.5	33.3 ± 8.6	<0.001
Diabetes, *n* (%)	0 (0)	74 (86)	—
Hypertension, *n* (%)	0 (0)	60 (69.8)	—
Dyslipidemia, *n* (%)	0 (0)	29 (33.7)	—
Glucose (mmol/L)	4.68 ± 0.5	12.8 ± 6.5	<0.001
HbA1c (%)	5.1 ± 0.31	9.6 ± 2.3	<0.001
Triglyceride (mmol/L)	0.89 (0.62–1.2)	1.72 (1.39–2.51)	<0.001
Total cholesterol (mmol/L)	3.15 (0.88–4.94)	5 (3.67–5.47)	<0.01
HDL cholesterol (mmol/L)	1.2 ± 0.2	1.04 ± 0.2	<0.01
LDL cholesterol (mmol/L)	2.67 (1.99–3.65)	2.8 (2.2–3.6)	NS
SBP (mmHg)	130	133.9 ± 17	NS
DBP (mmHg)	80	72 ± 8	<0.001
Lipid-lowering drugs, *n* (%)	0 (0)	22 (25.6)	—
ACE inhibitors, *n* (%)	0 (0)	33 (38.4)	—
ARBs, *n* (%)	0 (0)	11 (12.8)	—
Diuretics, *n* (%)	0 (0)	16 (18.6)	—
Calcium channel blockers, *n* (%)	0 (0)	26 (30.2)	—
Insulin, *n* (%)	0 (0)	38 (44.2)	—
Metformin, *n* (%)	0 (0)	55 (64)	—

Values are mean ± standard deviation or median (25%–75%) percentiles; MetS: metabolic syndrome; BMI: body mass index; HDL: high density lipoprotein; LDL: low density lipoprotein; SBP: systolic blood pressure; DBP: diastolic blood pressure; ACE: angiotensin converting enzyme; ARBs: angiotensin receptor blockers; NS: not significant.

**Table 2:** Pentosidine, CML, and sRAGE levels in control subjects and patients with MetS.

	Control subjects *n* = 80	Patients *n* = 86	*P*
Pentosidine (pmol/mL)	53.15 (36.61–60.34)	211.21 (69.94–379.89)	<0.001
CML (pg/mL)	171.75 (81.03–298.06)	440.38 (383.98–601.36)	<0.001
sRAGE (pg/mL)	117.33 (104.38–158.04)	155 (126.34–240.4)	0.014

Values are median (25%–75%) percentiles; CML: N (carboxymethyl) lysine; MetS: metabolic syndrome; sRAGE: soluble receptor for AGE.

**Table 3:** Treatment effect of metformin on pentosidine and CML levels in patients with MetS.

	Treated patients *n* = 55	Untreated patients *n* = 31	*P*
Pentosidine (pmol/mL)	140.72 (62.07–249.51)	372.45 (132.55–556.68)	0.01
CML (pg/mL)	504.39 (405.52–784.7)	439.76 (273.12–460.98)	0.083

Values are median (25%–75%) percentiles; CML: N (carboxymethyl) lysine.

**Table 4:** Correlation between pentosidine, CML, sRAGE levels and drugs treatment in patients with MetS: linear regression analysis.

Variables	Pentosidine		CML		sRAGE	
	*r*	*P*	*r*	*P*	*r*	*P*
Metformin treatment	−0.311	0.009	0.177	NS	0.401	<0.001
ACE inhibitors treatment	−0.054	NS	0.021	NS	0.352	0.002
Metformin + ACE inhibitors treatment	−0.071	NS	0.07	NS	0.408	<0.001

**Table 5:** Correlation between pentosidine, sRAGE, and drugs treatments in patients with MetS: multivariate regression analysis.

Variables	Pentosidine		sRAGE	
	*β*	*P*	*β*	*P*
Metformin treatment	−0.246	0.099	0.045	0.73
ACE inhibitors treatment	−0.299	0.182	0.08	0.685
Metformin + ACE inhibitors treatment	−0.236	0.951	0.326	0.143

ANOVA revealed a statistically not significant fit (*P* = 0.228).

A stepwise multivariate regression was performed.

## Abbreviations

AGEs: Advanced glycation end products
ACE: Angiotensin converting enzyme
CML: Carboxymethyl-lysine
MetS: Metabolic syndrome
sRAGE: Soluble receptor for advanced glycation end products
esRAGE: Endogenous secretory RAGE
MG: Methylglyoxal
ELISA: Enzyme-linked immunosorbent assay.
